# Supplementary material for: Complete vertebrate mitogenomes reveal widespread repeats and gene duplications
Source: Genome Biol. 2021 Apr 29;22:120. doi: 10.1186/s13059-021-02336-9 (PMC8082918; doi:10.1186/s13059-021-02336-9)
Supplement: Supplementary file 1 — Additional file 1: Fig. S1. Outline of the mitoVGP assembly pipeline. Fig. S2. Assembly success by the availability of long mtDNA reads. Fig. S3. PacBio CLR mitochondrial read counts in different tissues. Fig. S4. mitoVGP assembly results and comparisons. Fig. S5. Correlation between differences in repeat content and assembly length between the mitoVGP versus the Genbank/Refseq assemblies. Fig. S6. Paired comparisons of GC content between the mitoVGP assemblies and their Genbank/RefSeq counterparts. Fig. S7. Heatmap of k-mer-based sequence similarity of repetitive elements. Fig. S8. Read length deviation from the reference in kbp. Fig. S9. Long accurate HiFi reads from the VGP human trio mapped to the child mitogenome assembly. [file 13059_2021_2336_MOESM1_ESM.docx]

**Supplementary figures**


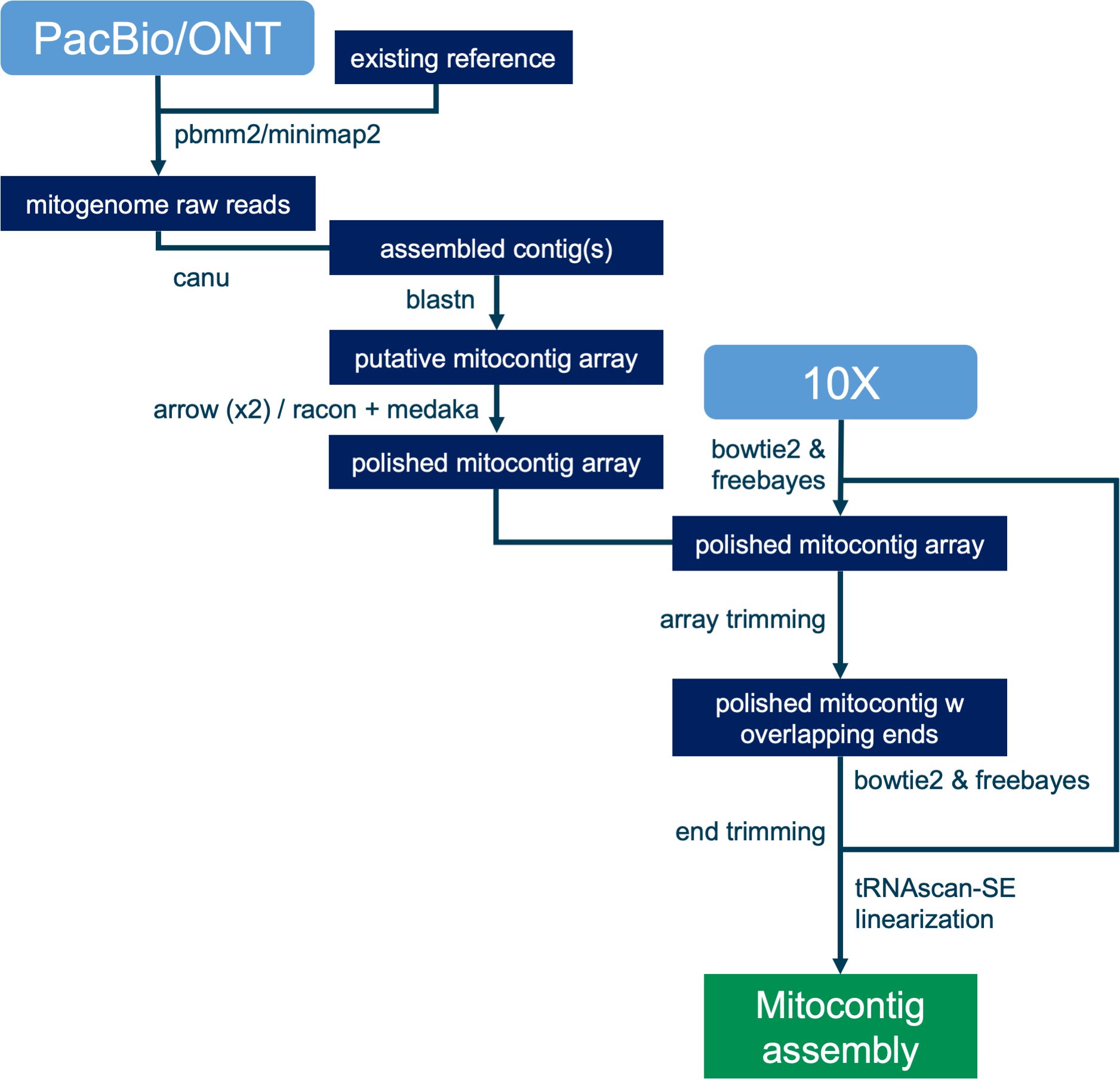


**Fig. S1 | Outline of the mitoVGP assembly pipeline.** Raw read data are represented by light blue rectangles. The outputs of each step of the workflow are represented by dark blue rectangles. Tools automatically employed at each step are represented next to the arrows in the flow.


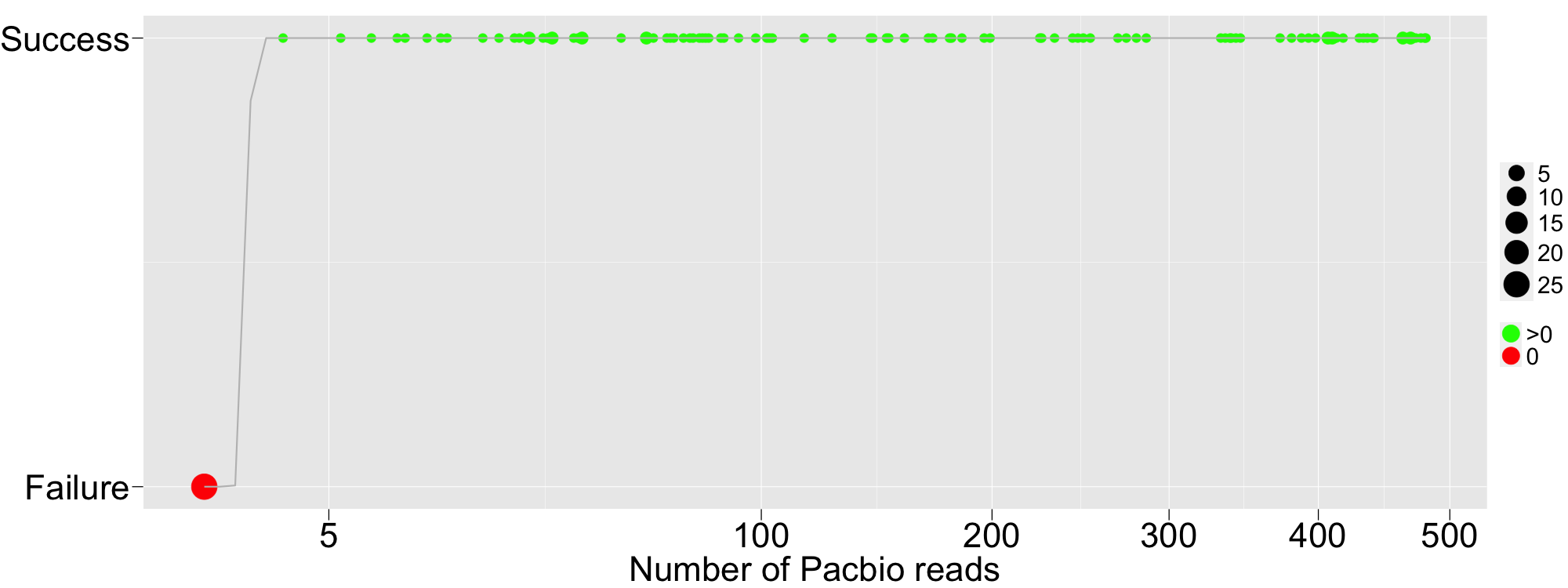


**Fig. S2 | Assembly success by the availability of long mtDNA reads.** The x axis is in sqrt to highlight success rate in the lower ranger. 0 read counts highlighted in red, >0 in green. The fitted curve corresponds to a generalized linear model Success ~ Number of long mtDNA reads using a binomial distribution.


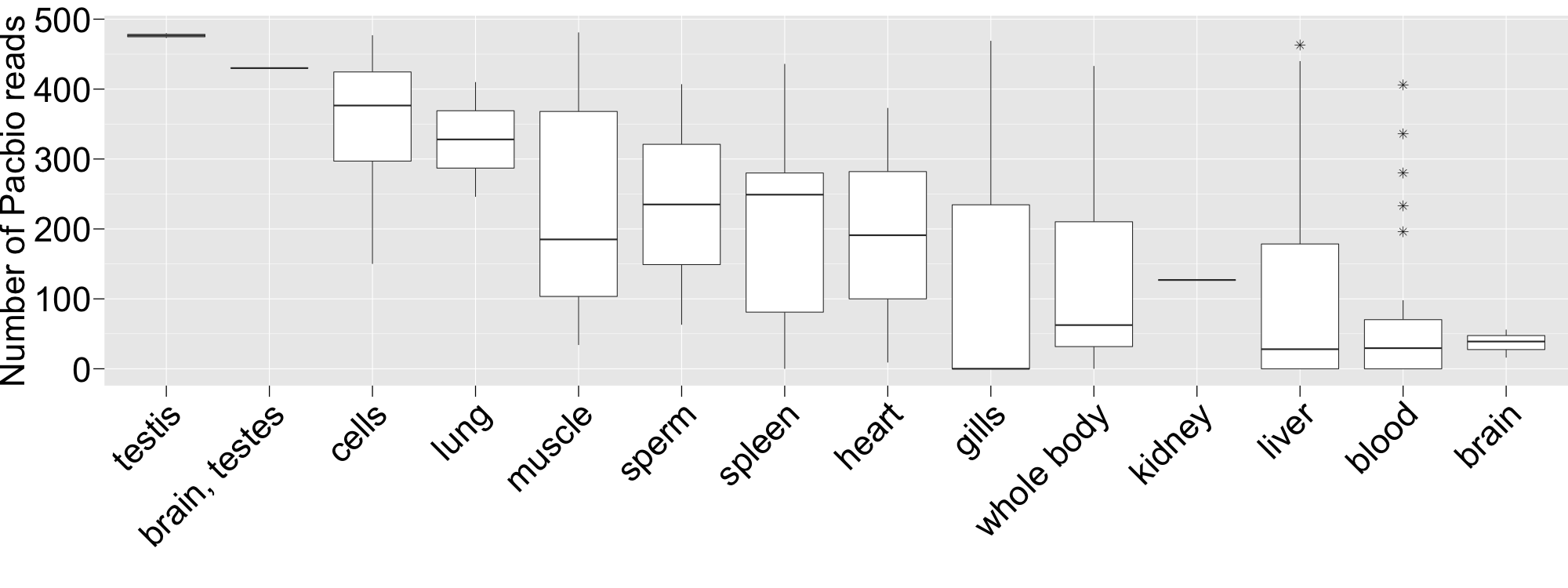


**Fig. S3 | PacBio CLR mitochondrial read counts in different tissues for all samples.** Black bar, average. Hinges correspond to the first and third quartiles. Whisker extends from the hinge to the values no further than 1.5 x IQR from the hinges. N = 100. *, outliers. Note that only groups with at least N = 3 were used for the statistical analyses.


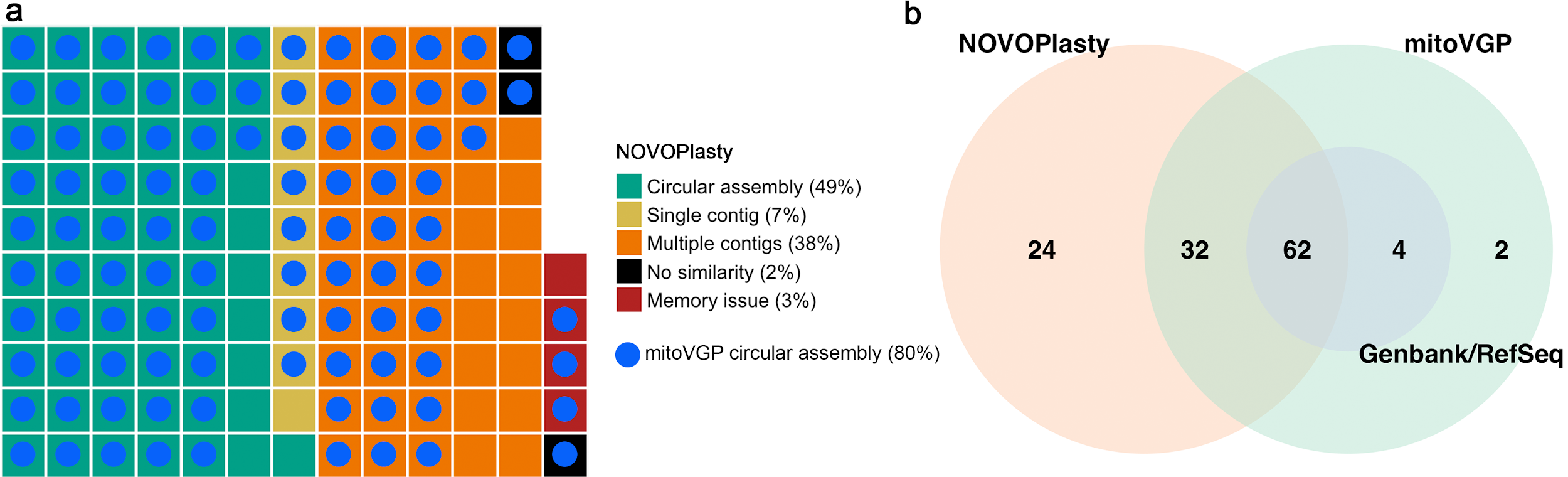


**Fig. S4 | mitoVGP assembly results and comparisons. a,** Benchmarking of the mitoVGP results with the short-read organelle genome assembler NOVOplasty. Of the successful assemblies, 49% (n = 61) of NOVOPlasty assembled as circular according to the software, 7% (n = 9) in a single contig (yellow), and 38% (n = 48) assembled in multiple contigs (orange). About 2% (n = 3) of the assemblies showed little or no similarity with corresponding reference and mitoVGP assemblies and were excluded from the analyses (black). The assembly failed in 3% (n = 4) of cases due to reproducible software errors (red). Matching successful mitoVGP assemblies are highlighted by the blue circles. **b,** Venn diagram of successful assemblies in the three datasets.


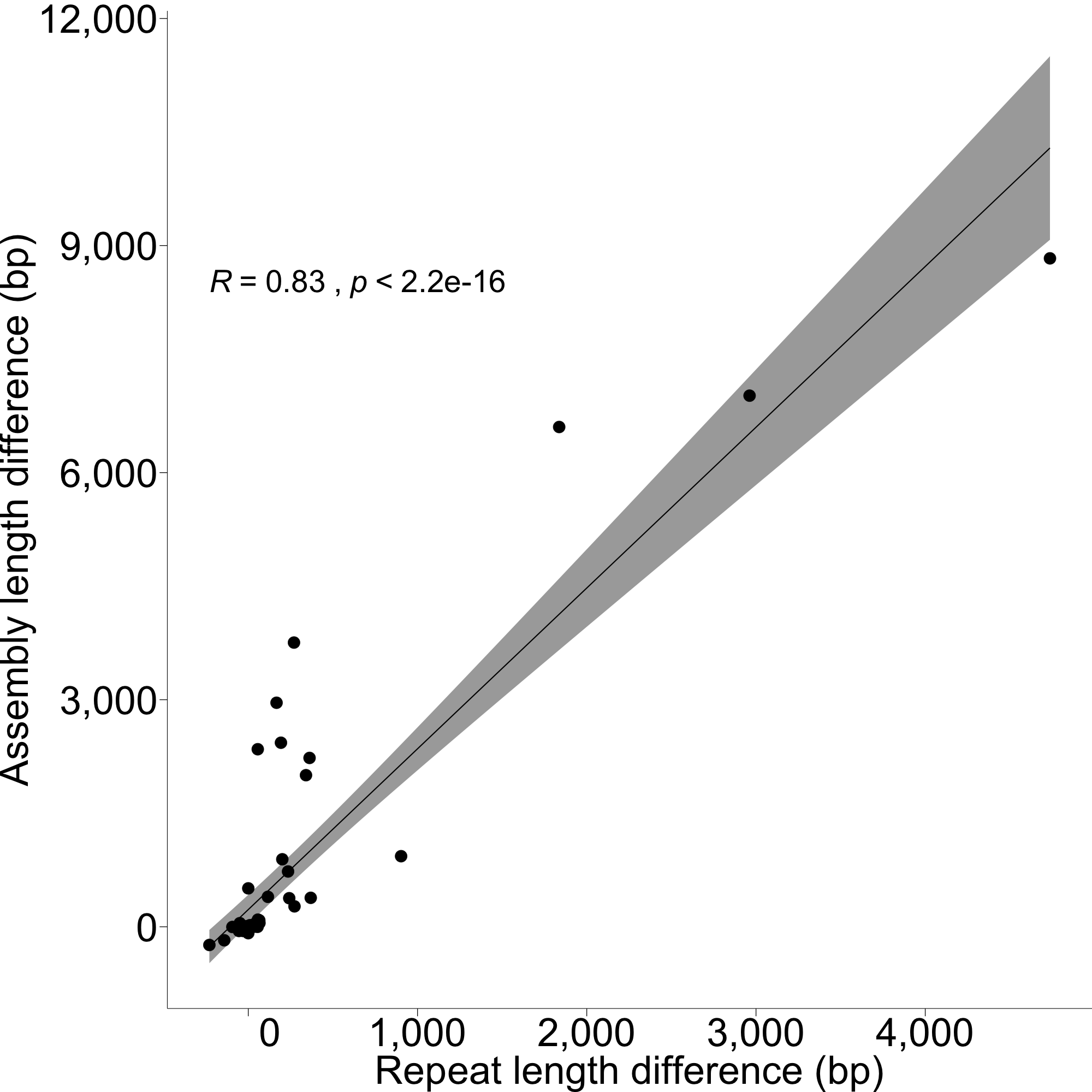


**Fig. S5 | Correlation between differences in repeat content and assembly length between the mitoVGP versus the Genbank/Refseq assemblies.** Regression line and confidence intervals are shown. Statistics: Spearman ρ correlation.


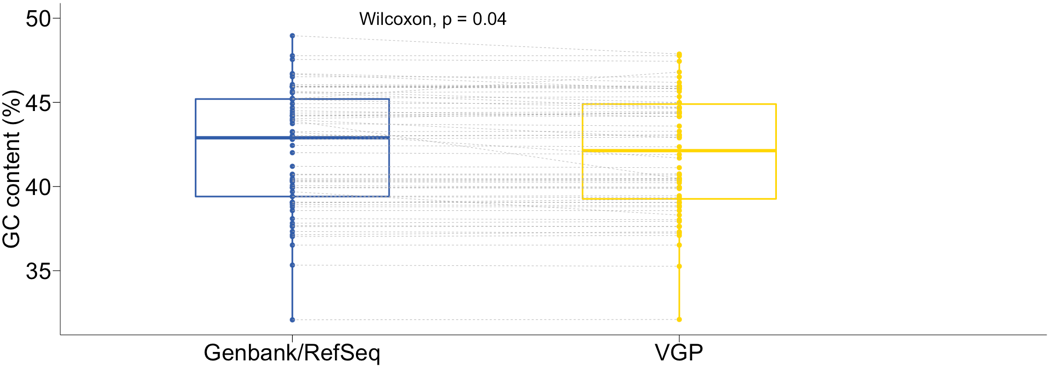


**Fig. S6 | Paired comparisons of GC content between the mitoVGP assemblies and their Genbank/RefSeq counterparts**. On average, the GC content was slightly lower in the mitoVGP assembly compared to the Genbank/REfSeq counterpart. Two-sided Wilcoxon test.


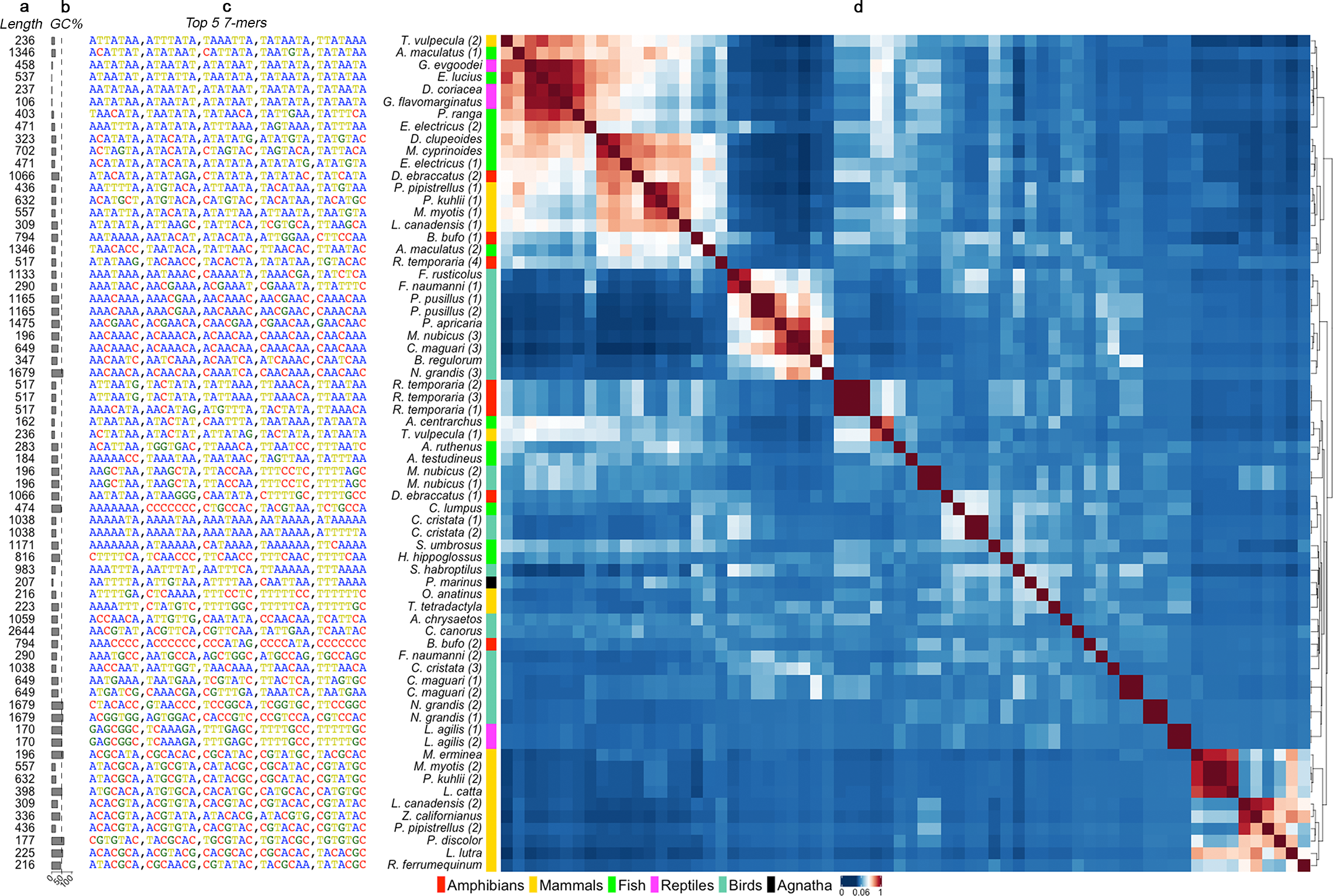


**Fig. S7 | Heatmap of k-mer-based sequence similarity of repetitive elements.** **a**, total repeat sequence length. **b**, % repeat GC-content. **c**, Top 5 most frequent 7-mers in the repeat. **d**, Sequence similarity clustering of repeats. When multiple repeats are present in the same assembly they are indicated with a progressive number.


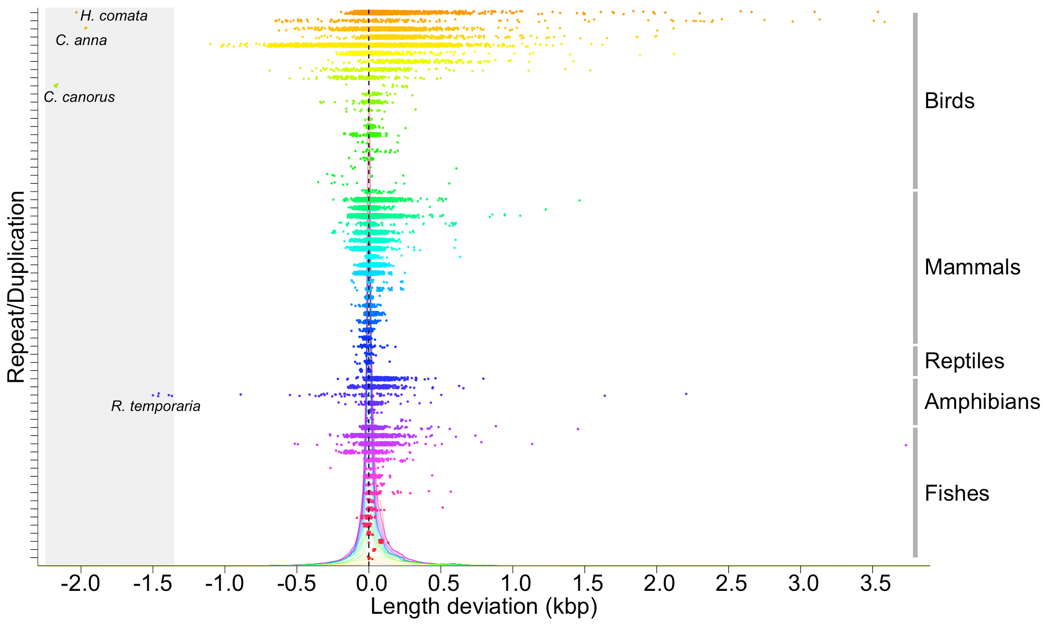


**Fig. S8 | Length deviation from the reference in kbp for each read spanning the repeat region.** No deviation from the assembled VGP reference is marked by the dashed line. Colors correspond to different repetitive and duplicated elements. Individual density distributions are shown in the background. The gray shaded area highlights four species that had reads that lack gene duplications when these are present in the mitoVGP assembly, suggesting potential heteroplasmy.

**
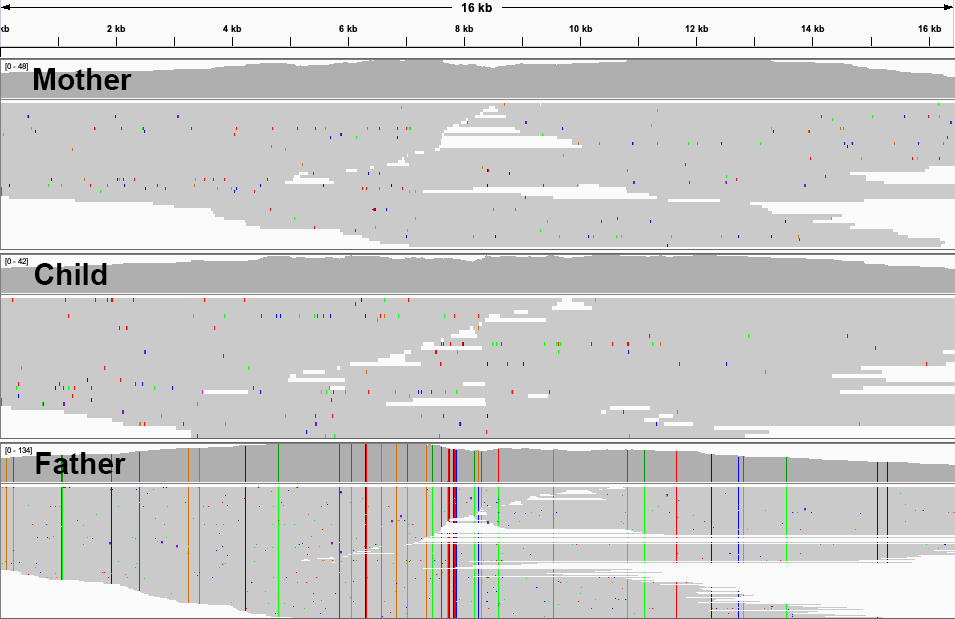
**

**Fig. S9 | Long accurate HiFi reads from the VGP human trio mapped to the child mitogenome assembly.** MtDNA CLR alignments in the mitoVGP human reference, based on trio data, showing uniform long-read coverage across the reference, highlighting inheritance patterns from the mother. First window, mother. Second window, child. Third window, father. Indels < 5 bp are masked.
