# Supplementary material for: Complete vertebrate mitogenomes reveal widespread repeats and gene duplications
Source: Genome Biol. 2021 Apr 29;22:120. doi: 10.1186/s13059-021-02336-9 (PMC8082918; doi:10.1186/s13059-021-02336-9)
Supplement: Supplementary file 8 — Additional file 8: Supplementary Note 1. Relationship between mtDNA sequencing, coverage and genome size. [file 13059_2021_2336_MOESM8_ESM.docx]

**Supplementary Note 1**

Given the original ratio between mtDNA and nDNA ΔL, the relative increase in the size of the nuclear genome length as well as in the total amount of raw data to attain the same coverage ΔS, the relative gain in the number of mtDNA reads ΔR_M_ is given by the formula:


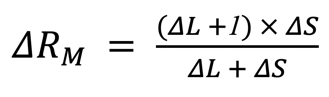


For example, assuming a typical vertebrate mitogenome size of 17 kbp, a mitochondria-rich tissue with 1,000 mitochondria per cell, when sequencing a nuclear genome three times bigger at the same coverage (e.g. a mammalian genome vs a bird genome) the expected relative gain in mtDNA reads is only 1.3%. Given that mitochondria-rich tissues would also invariably generate abundant mtDNA reads, this small difference clearly points to factors other than the total raw Gbp in the dataset, including the accuracy in estimating the nuclear genome size, predominantly driving the abundance of mtDNA reads.
