# Supplementary material for: Complete vertebrate mitogenomes reveal widespread repeats and gene duplications
Source: Genome Biol. 2021 Apr 29;22:120. doi: 10.1186/s13059-021-02336-9 (PMC8082918; doi:10.1186/s13059-021-02336-9)
Supplement: Supplementary file 12 — Additional file 12. Review history. [file 13059_2021_2336_MOESM12_ESM.docx]

Review History

**First round of review**

**Reviewer 1**

**Are you able to assess all statistics in the manuscript, including the appropriateness of statistical tests used?**

Yes, and I have assessed the statistics in my report.

**Comments to author:**

The paper "Complete vertebrate mitogenomes reveal widespread gene duplications and repeats" by Formenti et al. reports a pipeline that led to successful complete mitogenome assemblies of 100 vertebrate species of the Vertebrate Genomes Project (VGP). The information provided demonstrates, in my opinion, that mitoVGP, a new mitogenome assembler combining long and short reads, can indeed successfully assemble high-quality mitogenomes in a variety of datasets. The number, quality and variety of complete mitogenome assemblies and datasets presented allows a much more accurate comparison of sequence data and assembly strategies than ever before.

Taking into account the high quality of the analyses and the importance of the results, the extensive amount of data on which are based the conclusions, and the impact that they will have on a broad readership encompassing many areas of Life Science, I consider this paper perfectly appropriate for Genome Biology. Indeed, I am convinced that it will be very influential and frequently cited for a long time in the future.

Following is a list of minor issues to be addressed that would improve the clarity of the paper:

1. Title. It sounds a little bit ambiguous to me. As it is written "Gene" could refer to both duplications and repeats. You might consider the following: "Complete vertebrate mitogenomes reveal widespread repeats and gene duplications".

2. Key words. The first listed key word is "mitochondrion". Did you want to say "mitogenome" or better "mitochondrial DNA" considering that "mitogenome" is mentioned in the title?

3. Text. "Repeat regions" vs "Repetitive regions". Both terms are used multiple times in the text. Is the meaning the same (as I always thought) or different? If it is the same, I would use only one form to avoid misunderstandings.

4. Page 5, lines 116-118. Is the employed gene nomenclature (cob, coi, nad6) correct? Please double check this issue (also elsewhere; examples: figure 2 legend, line 285, Figure 3, etc.). For instance, MT-CYB or CYB is used for the cytochrome b gene in many organisms (human (see MITOMAP), mammals, chicken) I am familiar with.

5. Page 6, lines 130-131. "NUMTs originate from the partial or complete transposition of the mtDNA …". "By complete transposition" do you mean that there is published evidence that an entire mitogenome can be integrated as a single NUMT sequence? If so, provide the reference (Mishmar et al. 2004 do not say so); if not, please rephrase the sentence to avoid possible misinterpretations.

6. Line 155. "Lead" or "led"?

7. Supplementary tables. Double check the order of appearance in the text. Tables S4 and S5 appear in the text earlier than Table S3, and Table S9 earlier than Table S8.

8. Lines 175-176. "To test whether …, we tested whether …". Reword.

9. Fig. S3. The second column is associated with both "brain, testes" (and Table S5, 1 sample). Is it correct?

10. Lines 187-188. Double check the reported numbers (both N values and percentages); they are not the same of those listed in Table S5.

11. Lines 231-232. Please double check all numbers reported in the two lines relative to the percentage values reported in Fig. S4 and its legend. They do not appear to fit each other. Example, "of the successful assemblies (in the text 118 out of 125) …, 7% in a single contig (but 70 out of 125 in the text).

12. Line 243. I do no see any "yellow" in Fig. 1a.

13. Lines 261-262. Fig. 1h should be Fig. 1g and Fig. 1g should be F1g.1h.

14. Lines 275-278. The top three outliers … were found in birds. However, in Fig. 1e the second top outlier is Rana temporaria. Reword the sentence.

15. Lines 347-348. … always showing one more more repeats …?

16. Line 357. "… gene duplication event". Perhaps it is more correct "duplication event".

17. Line 387. Are there "genes" in the CR (control region)? No, as far as I know. Please reword.

**Reviewer 2**

**Are you able to assess all statistics in the manuscript, including the appropriateness of statistical tests used?**

Yes, and I have assessed the statistics in my report.

**Comments to author:**

Complete vertebrate mitogenomes reveal widespread gene duplications and repeats

Summary and overall impression:

The authors report a new assembly pipeline for mitogenomes (mitoVGP) that takes advantage of multiple sequencing technologies. They demonstrate the robustness of this pipeline by applying it to 100 vertebrate species from the Vertebrate Genomes Project (VGP) and find evidence of previously undescribed repeats and gene duplications.

Overall the pipeline has an 80% success rate and I am impressed with the author's exploration of assembly failures and have no improvements to suggest. Additionally the authors provide more than sufficient evidence demonstrating the quality of the assemblies. I believe that the new repeats are likely genuine and not sequencing or assembly artifacts.

In my specific comments I point out a few areas where I would like to see further analysis of the genic repetitive content, but my overall opinion of the manuscript is positive and my comments minor. Moreover, I think the assemblies provided here will be of considerable practical value to the field.

Specific comments for the authors:

1) The authors remark that NUMTs complicate short read assembly of mitochondrial sequences but it seems to me that they could also complicate long-read assemblies. In light of this how does mitoVGP filter out or tag NUMTs during the assembly process? I found one mention in the methods section about the possibility of filtering on read length but it was unclear to me if this filter was applied. If all 100 mitoVGP assemblies are circular then this is a non-issue since those would not be NUMTs; however, from the text I am not certain that the assemblies are indeed circular.

2) It is hard for me to interpret Figure 3b because its scale is absolute instead of relative. The authors observe a positive length deviation and hypothesize that is due to PacBio's increased frequency of insertions vs deletions; however, without knowing the size of the sequence being compared it is hard to interpret the kbp differential. For example, a 1.0 kbp difference across 16 kbp could reasonably be attributed to sequencing error; however, a 1.0 kbp difference across 4 kbp of sequence seems highly unlikely if the cause is sequencing error. I suggest the authors change the scale to show the fractional difference in length, or include an equivalent analysis elsewhere. Additionally, an indication of whether an observed deviation is thought to be due to PacBio sequencing error or heteroplasmy would be useful, as well as a quantification of the expected degree of heteroplasmy.

3) The authors highlight several examples of complete or partial gene duplications, but do not state how many of the 68 repeat regions contain complete gene duplications vs partial. A table with a more detailed breakdown of partial and complete gene duplications and the species they are in would add impact to the work in my opinion.

4) Figure 4 shows an interesting result, but is in my opinion underexplored. The authors comment on the multimodal distribution in VGP assemblies but do not explore the extra sequence beyond saying that the extra peaks are due to repeats and duplications. The multimodal distribution would indicate to me that some duplicated elements are recurrently incorporated across different assemblies. Specifically, I would like to know if the second peak is largely caused by one particular duplication event, and if that duplication event contains full or partial gene copies.

5) For lines 448-450, would it be more accurate to say that you estimate that 15% of complete vertebrate mitogenomes are incorrectly assembled in Genban/RefSeq since there was no analysis of other mitogenomes outside of vertebrates (to my knowledge)?

6) The data and code are linked and described appropriately with the exception of line 857 which contains an invalid or incorrectly linked url that leads to a 404 error.

**Authors Response**

**Point-by-point responses to the reviewers’ comments:**

Reviewer #1

1. Title. It sounds a little bit ambiguous to me. As it is written "Gene" could refer to both duplications and repeats. You might consider the following: "Complete vertebrate mitogenomes reveal widespread repeats and gene duplications".

*Response: We agree with the reviewer and we have modified the title according to the suggestion.*

2. Key words. The first listed key word is "mitochondrion". Did you want to say "mitogenome" or better "mitochondrial DNA" considering that "mitogenome" is mentioned in the title?

*Response: We replaced ‘mitochondrion’ with ‘mitochondrial DNA’.*

3. Text. "Repeat regions" vs "Repetitive regions". Both terms are used multiple times in the text. Is the meaning the same (as I always thought) or different? If it is the same, I would use only one form to avoid misunderstandings.

*Response: Yes, we consider them to have the same meaning. We have adopted ‘repetitive regions’ throughout the text.*

4. Page 5, lines 116-118. Is the employed gene nomenclature (cob, coi, nad6) correct? Please double check this issue (also elsewhere; examples: figure 2 legend, line 285, Figure 3, etc.). For instance, MT-CYB or CYB is used for the cytochrome b gene in many organisms (human (see MITOMAP), mammals, chicken) I am familiar with.

*Response: Following reviewer's suggestion, we have updated the gene nomenclature throughout the text, figures and table using names approved by the HGNC for genes and by VGNC for proteins (e.g. nad1 → MT-ND1 for mitochondrially encoded NADH dehydrogenase 1).*

5. Page 6, lines 130-131. "NUMTs originate from the partial or complete transposition of the mtDNA …". "By complete transposition" do you mean that there is published evidence that an entire mitogenome can be integrated as a single NUMT sequence? If so, provide the reference (Mishmar et al. 2004 do not say so); if not, please rephrase the sentence to avoid possible misinterpretations.

*Response: Yes, this is what we meant. We revised from “complete” to “near complete”. We now cite Dayama et al., 2014 as an example in the introduction.*

6. Line 155. "Lead" or "led"?

*Response: Typo corrected in text.*

7. Supplementary tables. Double check the order of appearance in the text. Tables S4 and S5 appear in the text earlier than Table S3, and Table S9 earlier than Table S8.

*Response: We corrected the table numbering according to their appearance in the text.*

8. Lines 175-176. "To test whether …, we tested whether …". Reword.

*Response: Fixed.*

9. Fig. S3. The second column is associated with both "brain, testes" (and Table S5, 1 sample). Is it correct?

*Response: Yes, unfortunately this a single pooled sample. We kept both tissue names in the figure for completeness. However, only groups with N>3 were considered in the statistical analyses, as detailed in the methods section. This is now mentioned in the figure legend.*

10. Lines 187-188. Double check the reported numbers (both N values and percentages); they are not the same of those listed in Table S5.

*Response: We thank the reviewer for catching this, which we now corrected. The table reported the correct numbers, and the statistical analyses and figures were based on these numbers. However, they were not initially corrected from an earlier version of the main text.*

11. Lines 231-232. Please double check all numbers reported in the two lines relative to the percentage values reported in Fig. S4 and its legend. They do not appear to fit each other. Example, "of the successful assemblies (in the text 118 out of 125) …, 7% in a single contig (but 70 out of 125 in the text).

*Response: We thank the reviewer for pointing this out. We rechecked Fig. S4; the caption and the text and they all match. The confusion may have originated by the presence of 9 NOVOPlasty assemblies (7%) that are indeed assembled in a single contig; however, they contain long overhangs, and are highlighted separately in the figure and in the text. To better highlight that numbers match, we report the absolute numbers of assemblies next to the percentage in the Fig. S4 caption.*

12. Line 243. I do not see any "yellow" in Fig. 1a.

*Response: The yellow is almost invisible due to expected full overlap with the boxes representing the other categories. We clarified this in the Fig. 1a caption.*

13. Lines 261-262. Fig. 1h should be Fig. 1g and Fig. 1g should be F1g.1h.

*Response: Corrected in text.*

14. Lines 275-278. The top three outliers … were found in birds. However, in Fig. 1e the second top outlier is Rana temporaria. Reword the sentence.

*Response: Sentence fixed.*

15. Lines 347-348. … always showing one more more repeats …?

*Response: Fixed.*

16. Line 357. "… gene duplication event". Perhaps it is more correct "duplication event".

*Response: Fixed according to the proposed suggestion.*

17. Line 387. Are there "genes" in the CR (control region)? No, as far as I know. Please reword.

*Response: Reworded with deleting mentioning the CR.*

Reviewer #2

1) The authors remark that NUMTs complicate short read assembly of mitochondrial sequences but it seems to me that they could also complicate long-read assemblies. In light of this how does mitoVGP filter out or tag NUMTs during the assembly process? I found one mention in the methods section about the possibility of filtering on read length but it was unclear to me if this filter was applied. If all 100 mitoVGP assemblies are circular then this is a non-issue since those would not be NUMTs; however, from the text I am not certain that the assemblies are indeed circular.

*Response: Table S1 contains the code to replicate all the assemblies, including the filtering options that were used for each individual assembly. Specifically, read length filters were applied in 19 cases using mitoVGP -f option. We have added this information also to the relative methods section for all important options mentioned in the methods (-f, -o, -p, -z).*

*To clarify, mtDNA reads in the library prep range from relatively short reads (<1,000 bp) to full-length reads (16,000-23,000 bp). The latter occurs when a full-length linearized mitogenome has SMRTbell adapters ligated to it. The separation of NUMT reads from mtDNA reads is done with the assembler (Canu), and is based on the consistency of the overlaps in the assembly graph. Canu filters reads below 1kb by default, so any NUMT reads below that length will not contribute to the graph. If a longer NUMT read also contains sequence from the nuclear genome, this is also not expected to contribute to the final mitogenome sequence when the graph is resolved because the overlap will not be consistent. Indeed, Canu usually labels the putative mtDNA assembly as REPEAT owing to the fact that the assembly graph is circular. However, long NUMT reads may confuse the assembler because they will determine multiple edges or paths in the assembly graph. These usually result in assembly failure. We have therefore introduced the -f option to filter out upfront any read longer than a plausible mitogenome length (e.g. 25kb), and we clarified this further in the methods section.*

2) It is hard for me to interpret Figure 3b because its scale is absolute instead of relative. The authors observe a positive length deviation and hypothesize that is due to PacBio's increased frequency of insertions vs deletions; however, without knowing the size of the sequence being compared it is hard to interpret the kbp differential. For example, a 1.0 kbp difference across 16 kbp could reasonably be attributed to sequencing error; however, a 1.0 kbp difference across 4 kbp of sequence seems highly unlikely if the cause is sequencing error. I suggest the authors change the scale to show the fractional difference in length, or include an equivalent analysis elsewhere. Additionally, an indication of whether an observed deviation is thought to be due to PacBio sequencing error or heteroplasmy would be useful, as well as a quantification of the expected degree of heteroplasmy.

*Response: As suggested by the reviewer, we have provided a revised Figure 3b where the scale is expressed relative to the length of the region in the reference. We provide the original figure where the absolute length deviations in kbp are reported as Fig. S8. We have also highlighted in the main text as Fig. 4 our example of precise quantification of heteroplasmy in the case of the Kakapo with an 84 bp repeat. Here, the mtDNA read depth is sufficient to see deviations from the 11-repeat of this 84 bp allele to minor alleles with 6 to 14 repeats of the 84 bp.*

3) The authors highlight several examples of complete or partial gene duplications, but do not state how many of the 68 repeat regions contain complete gene duplications vs partial. A table with a more detailed breakdown of partial and complete gene duplications and the species they are in would add impact to the work in my opinion.

*Response: We have added the information as to whether the duplication was complete or partial to Table S1 - columns AL, where the detail on the duplications is reported next to the information on repeats for each species. When we do not report the length of the partial duplication, it is assumed to be complete. We based our decision on mitos2 gene annotations, and considered complete any duplication comparable in length for at least >95%, which we now include in the manuscript. This is now mentioned in the methods.*

4) Figure 4 shows an interesting result, but is in my opinion underexplored. The authors comment on the multimodal distribution in VGP assemblies but do not explore the extra sequence beyond saying that the extra peaks are due to repeats and duplications. The multimodal distribution would indicate to me that some duplicated elements are recurrently incorporated across different assemblies. Specifically, I would like to know if the second peak is largely caused by one particular duplication event, and if that duplication event contains full or partial gene copies.

*Response: We have now added information on the relative contribution of taxa and type of duplication events in the main text. Specifically, the general divergence pattern for lengths > 18 kb is contributed by duplications and repeats in non-mammalian species, and the second peak is contributed by a duplication involving MT-CYB, MT-TT, MT-TP, MT-ND6 and MT-TE genes that we have frequently observed in birds (N = 9), and which is usually missed in the short read assemblies. MT-CYB is usually the only gene partially duplicated, and the length of the duplication can vary (120-609bp). This may be due to a positional factor, with Cob being at the beginning of the duplicated region. We now mention this finding in the text. As previously stated, duplicated genes are detailed in Table S1 column AL, and now whether the duplication is complete or partial is also reported.*

5) For lines 448-450, would it be more accurate to say that you estimate that 15% of complete vertebrate mitogenomes are incorrectly assembled in Genbank/RefSeq since there was no analysis of other mitogenomes outside of vertebrates (to my knowledge)?

*Response: Yes, this is correct and we now specifically state vertebrates.*

6) The data and code are linked and described appropriately with the exception of line 857 which contains an invalid or incorrectly linked url that leads to a 404 error.

*Response: We have now updated the link.*
